# The Enigma of Idiopathic Multicentric Castleman Disease: An Elusive Diagnosis

**DOI:** 10.7759/cureus.73156

**Published:** 2024-11-06

**Authors:** Jessica Ohemeng-Dapaah, Afoma Onyechi, Ayesha Kang, Alexandre Lacasse, Jyotsana Sinha

**Affiliations:** 1 Internal Medicine, Sisters of Saint Mary (SSM) Health St. Mary’s Hospital, St. Louis, USA; 2 Hematology and Medical Oncology, Sisters of Saint Mary (SSM) Health St. Mary’s Hospital, St. Louis, USA

**Keywords:** castleman disease, histopathology, inflammation, lymphadenopathy, multicentric, pancytopenia, plasmacytosis, siltuximab

## Abstract

Castleman disease (CD) is a rare lymphoproliferative disorder encompassing a spectrum of conditions with distinct histopathological findings and varied clinical presentations. Diagnostic challenges are often encountered due to overlapping features with other malignancies, infections, and autoimmune disorders. Idiopathic multicentric Castleman disease (iMCD) is a subtype of CD, characterized by generalized lymphadenopathy, polyclonal lymphoproliferation, systemic inflammation, and a cytokine storm that can be life-threatening. Here, we present a case of iMCD in a 70-year-old male with constitutional symptoms, dyspnea, and pancytopenia. Imaging demonstrated multifocal lymphadenopathy. Histopathological examination of a cervical lymph node revealed Castleman-like features, meeting the major criteria for a diagnosis of iMCD. Elevated interleukin-6 (IL-6) levels further supported the diagnosis. Treatment with siltuximab was planned but was preempted by the patient’s demise following acute heart failure exacerbation. Diagnosing CD necessitates a thorough evaluation to differentiate it from other diseases. Treatment strategies, particularly IL-6 blockade, play a crucial role in the management of iMCD and improve patient outcomes.

## Introduction

Castleman disease (CD) is an uncommon disease, with an estimated yearly incidence of 4,300 to 5,200 cases in the United States [1]. It is a collection of at least four conditions that share a spectrum of distinctive histopathological findings but differ in etiology, presentation, treatment, and outcome. The four main subtypes of CD are unicentric Castleman disease (UCD), human herpesvirus-8 (HHV-8)-associated multicentric Castleman disease (HHV-8+MCD), HHV-8-negative/idiopathic multicentric Castleman disease (iMCD), and POEMS (polyneuropathy, organomegaly, endocrinopathy, monoclonal plasma cell disorder, skin changes)-associated multicentric Castleman disease [1]. Although the exact cause of CD is yet unknown, it has been linked to immunological deficiencies, aberrant immune regulation, viral infection, and aberrant cytokine release [2]. Its varied clinical presentations and unpredictable course often pose diagnostic and treatment challenges. UCD refers to the involvement of a single lymph node or a single region of lymph nodes, whereas multicentric Castleman disease (MCD) involves the involvement of multiple lymph node regions [3,4]. iMCD has an estimated incidence of 5 per million person-years [5]. A diagnosis of iMCD requires multicentric lymphadenopathy with defined histopathology, specific clinical/laboratory changes, and the exclusion of other diseases that may mimic iMCD [6-8]. Due to its uncommon nature and wide range of clinical manifestations, diagnosing iMCD can be difficult and frequently calls for a thorough assessment that includes clinical, radiological, and histological evaluations. Management of iMCD remains a challenge [9]. The main treatment objectives are to control symptoms, lower systemic inflammation, and halt the disease’s progression [10,11]. Here, we present a case of iMCD in an elderly male who mainly presented with dyspnea and discuss the clinical manifestations, diagnostic challenges, and therapeutic considerations encountered in managing this rare and complex disorder.

## Case presentation

**Figure 1:**
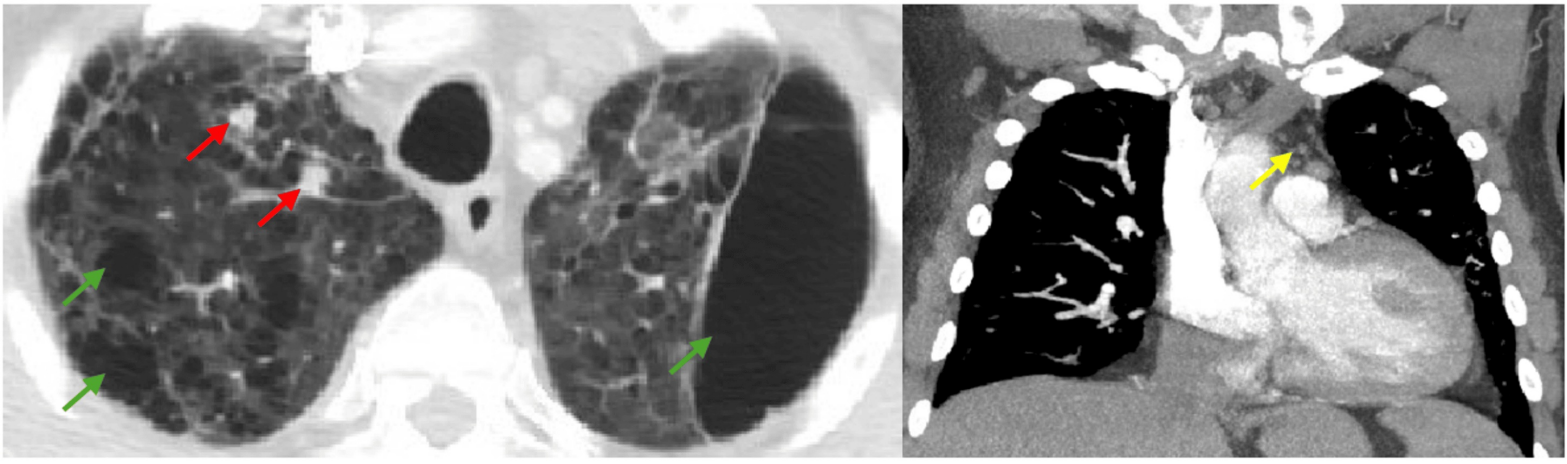
Images from the chest CT showing markedly emphysematous lungs (green arrows), pulmonary nodules (red arrows), and mediastinal lymphadenopathy (yellow arrow).

A 70-year-old male with a past medical history of hypertension, deep vein thrombosis, lung emphysema following COVID-19 pneumonia, and heart failure with moderately reduced ejection fraction presented to the emergency department with dyspnea. He was mildly confused, febrile, tachycardic, and tachypneic. A chest computed tomography (CT) scan showed markedly emphysematous lungs, patchy interstitial infiltrates, pulmonary nodules, and enlarged mediastinal lymph nodes (Figure 1).

**Figure 2:**
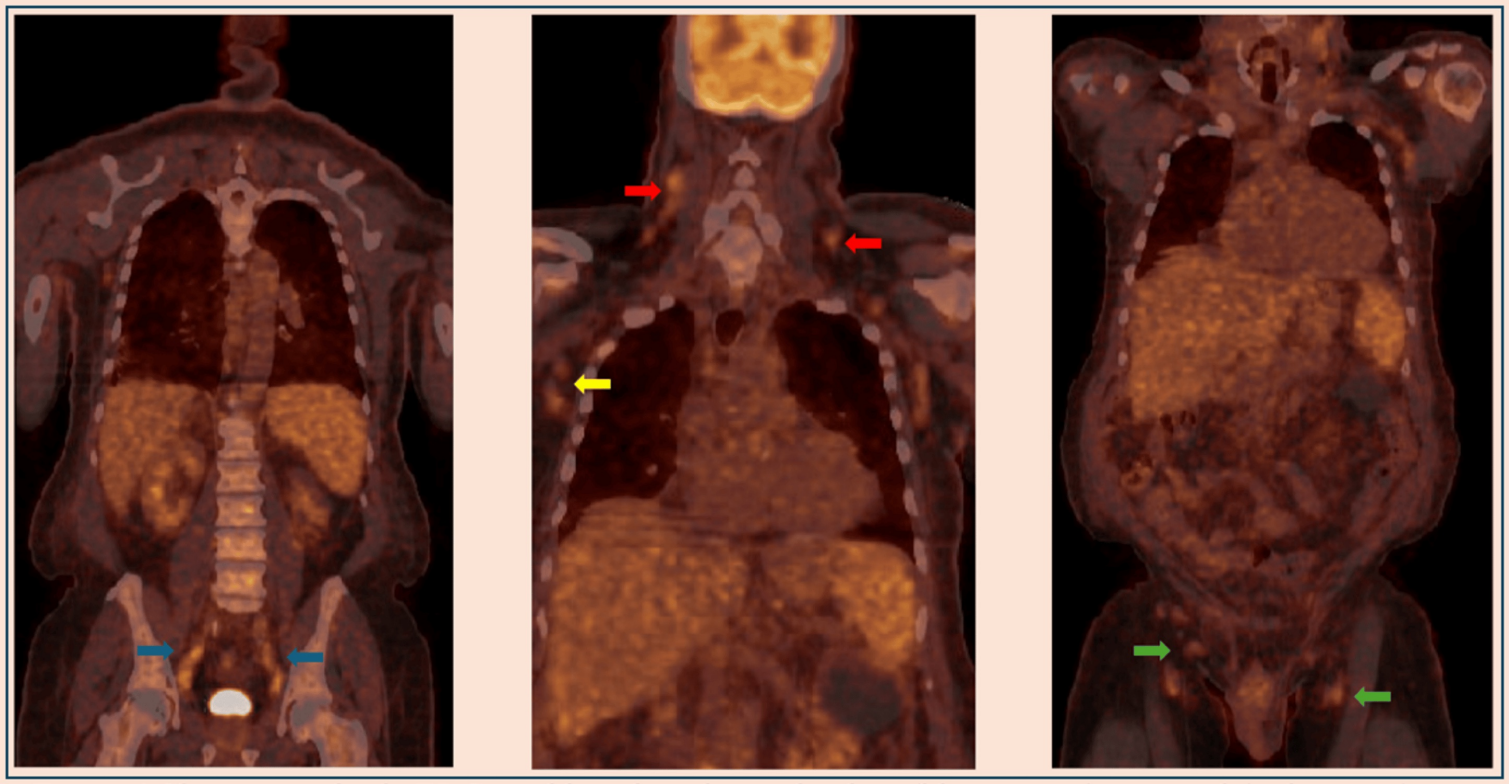
[18F]-fluorodeoxyglucose-avid lymph nodes in the iliac (blue arrows), cervical (red arrows), axillary (yellow arrow), and inguinal (green arrows) regions.

He was noted to have significant pancytopenia with a platelet count of 84 × 10^9^/L (normal range: 150-420 × 10^9^/L), anemia with a hemoglobin of 10.0 g/dL (normal range: 11.9-15.8 g/dL), and leukopenia with a white blood cell count of 2.9 × 10^9^/L (normal range: 4.0-10.7 × 10^9^/L). All blood counts were within normal limits five months prior. Evaluation of lactate dehydrogenase (LDH), reticulocyte count, and haptoglobin showed no evidence of hemolysis. Vitamin B12 was slightly decreased; folate and ferritin levels were normal; and inflammatory markers were elevated. Peripheral smear showed leukopenia, normocytic anemia, and thrombocytopenia with no blasts or immature cells seen. Intravenous antibacterial treatment was initiated for presumed sepsis due to pneumonia. Clinical improvement ensued and the patient was discharged home. Follow-up revealed persistent pancytopenia with fluctuating blood indices. Over the course of a few weeks, platelet count dropped to 36 × 10^9^/L and hemoglobin dropped as low as 6.5 g/dL. Human immunodeficiency virus, chronic viral hepatitis, and autoimmune screening tests (rheumatoid factor, lupus anticoagulant, antinuclear antibody) were negative. Serum protein electrophoresis showed polyclonal hypergammaglobulinemia. An M spike or features suggestive of a monoclonal process were not present. Bone marrow biopsy showed marked infiltration of plasma cells, displaying normal morphology, comprising ~50-60% of total cells, consistent with polytypic plasmacytosis. A positron emission tomography (PET)-CT scan showed multiple moderately [18F]-fluorodeoxyglucose (FDG)-avid lymph nodes above and below the diaphragm. Multiple bilateral cervical, axillary, and subpectoral lymph nodes with increased metabolic activity were seen. Multiple FDG-avid abdominal and pelvic lymph nodes involving the retroperitoneal, bilateral iliac regions, and bilateral inguinal lymph nodes were also seen (Figure 2). The lymph node sizes ranged from 0.8 cm to 1.3 cm. Finally, increased metabolic activity noted in the spleen and bone marrow was suspicious for tumor involvement.

**Figure 3:**
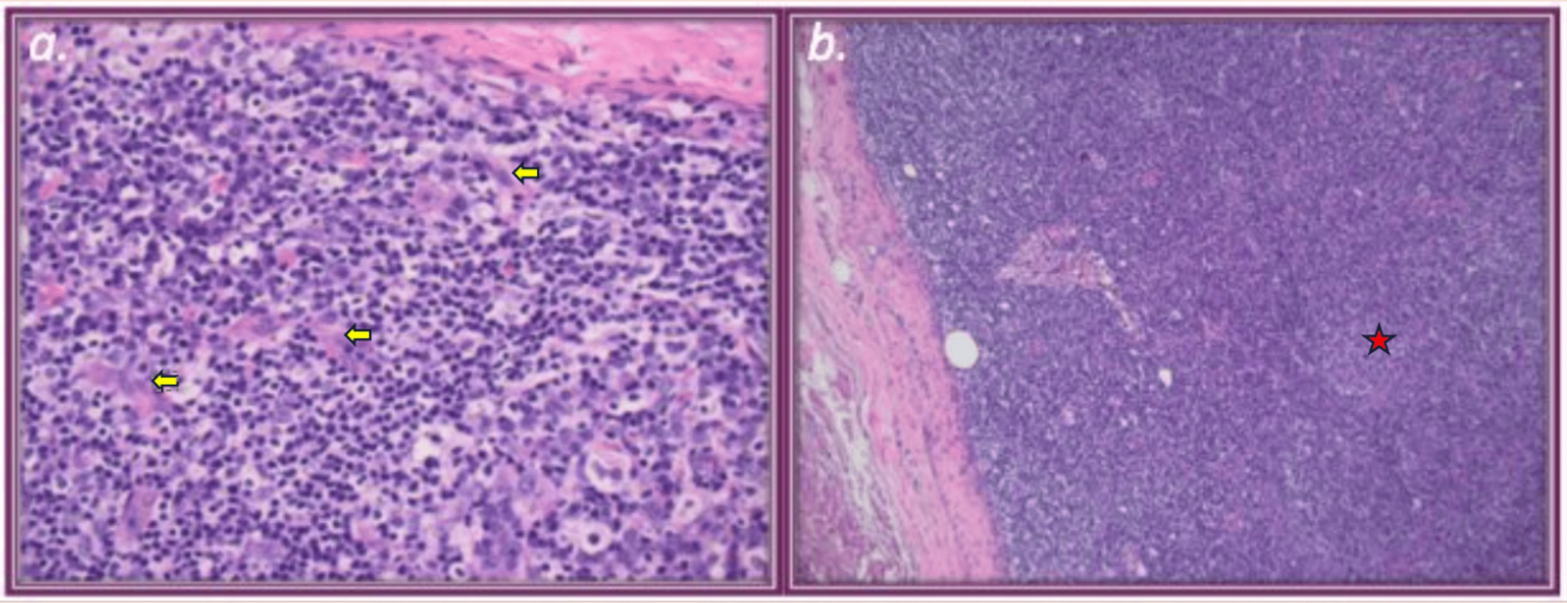
Lymph node slides with histopathologic features of Castleman disease. (a) Hematoxylin and eosin (H&E) 400×: interfollicular hypervascularity (yellow arrows) and plasmacytosis. (b) H&E 100×: interfollicular hypervascularity and atrophic follicle with few germinal centers (red star).

A biopsy of a cervical lymph node showed Castleman-like features, notably prominent interfollicular vascular proliferation and polytypic plasmacytosis. Retained lymph node organization and immunophenotypes did not favor T-cell lymphoma, specifically angioimmunoblastic T-cell lymphoma, but rather a response consistent with an immune dysregulation such as HHV-8-negative iMCD. Histopathologic analysis showed expansion of the paracortical and interfollicular areas by sheets of plasma cells (Figure 3a). Germinal centers appeared atrophic and were surrounded by plasma cells and prominent interfollicular vascularity (Figure 3b).

Interleukin-6 (IL-6) levels were elevated at 38.9 pg/mL (normal range: 0.0-13.0 pg/mL). A diagnosis of iMCD was made based on lymph node histopathologic features, lymph nodes ≥1 cm in diameter in ≥2 locations, as well as the presence of constitutional symptoms, splenomegaly, edema, anemia, thrombocytopenia, hypoalbuminemia, elevated inflammatory markers, and polyclonal hypergammaglobulinemia (Table 1).

**Table 1:** Minor laboratory criteria for the diagnosis of idiopathic multicentric Castleman disease. CRP: C-reactive protein; ESR: erythrocyte sedimentation rate; eGFR: estimated glomerular filtration rate

Minor laboratory criteria	Case patient laboratory values	Lab reference ranges
Elevated CRP (>10 mg/L) or ESR (>15 mm/hour)	ESR 61 mm/hour	ESR 0–20 mm/hour
Anemia (hemoglobin <12.5 g/dL for males, hemoglobin <11.5 g/dL for females)	Hemoglobin 6.5 g/dL	Hemoglobin 12.0–17.6 g/dL
Thrombocytopenia (platelet count <150 × 10^9^/L) or thrombocytosis (platelet count >400 × 10^9^/L)	Platelet count 36 × 10^9^/L	Platelet count 153–416 × 10^9^/L
Hypoalbuminemia (albumin <3.5 g/dL)	Albumin 1.5 g/dL	Albumin 3.4–5.0 g/dL
Renal dysfunction (eGFR <60 mL/minute/1.73m^2^) or proteinuria (total protein 150 mg/24 hour or 10 mg/100 mL)	eGFR 18 mL/minute/1.73m^2^	eGFR >90 mL/minute/1.73m^2^
Polyclonal hypergammaglobulinemia (total gamma-globulin or immunoglobulin G >1,700 mg/dL)	IgG 5,865 mg/dL	IgG 603–1,613 mg/dL

A negative latency-associated nuclear antigen-1 (LANA-1) immunohistochemistry stain of the lymph node ruled out HHV-8-associated MCD. Treatment with siltuximab was planned. Unfortunately, before therapy initiation, the patient succumbed to complications of acute heart failure.

## Discussion

Benjamin Castleman, director of the Department of Pathology at Massachusetts General Hospital, in 1954 first described CD as localized mediastinal lymph node enlargement with increasing numbers of lymphoid follicles, germinal center involution, and significant capillary proliferation, including follicular and interfollicular endothelial hyperplasia [1,12]. In UCD, lymph node enlargement is seen in a single region. Systemic symptoms typically resolve following surgical excision of involved lymph nodes [13,14]. Conversely, MCD is characterized by a cytokine storm, systemic inflammation, and polyclonal lymphoproliferation that may be life-threatening [7].

Since its founding in 2012, the Castleman Disease Collaborative Network (CDCN) has produced recommendations, diagnostic criteria, and expanded research on iMCD. The development of a three-part criterion that includes major criteria, minor criteria, and diseases to be excluded has substantially aided in the diagnosis and subsequent treatment of CD [7]. Many of the minor criteria were seen in our patient, including anemia, thrombocytopenia, elevated erythrocyte sedimentation rate, edema, and constitutional symptoms including fatigue. These findings remain non-specific. The clinical, histologic, and immunologic features of iMCD overlap with other malignancies, infections, and autoimmune disorders (Table 2).

**Table 2:** Diseases that mimic idiopathic multicentric Castleman disease.

Autoimmune diseases	Malignant/Lymphoproliferative diseases	Infections
Rheumatoid arthritis	Lymphoma	Epstein-Barr virus lymphoproliferative disease
Systemic lupus erythematosus	Multiple myeloma	Human herpesvirus-8-related multicentric Castleman disease
Adult-onset Still’s disease	Primary lymph node plasmacytoma	
Systemic juvenile idiopathic arthritis	Follicular dendritic cell sarcoma	

Therefore, iMCD remains a diagnosis of exclusion [15]. Ruling out these other diseases can take a considerable amount of time and lead to a delay in diagnosis. Strach et al. reported a case of iMCD in a patient who presented with fever, lymphadenopathy, and joint pain. An initial diagnosis of Still’s disease was made; however, the progression of symptoms despite treatment led to a diagnosis of iMCD seven years after the patient first reported symptoms [16].

According to Williams, mediastinal lymphadenopathy occurs in 70% of cases, followed by the neck and abdomen in 15% of cases. However, it can be anywhere, including the lungs, pelvis, and retroperitoneum [17]. In this case, the patient had multifocal lymph node enlargement involving the cervical, mediastinal, bilateral axillary, sub-pectoral, paratracheal, abdominal, pelvic, and retroperitoneal regions. Given this distribution and the histopathological features seen, we were confident in establishing a diagnosis of MCD. MCD is further classified based on the presence or absence of HHV-8 infection. HHV-8 infection is identified either by blood polymerase chain reaction or with a positive LANA-1 on immunohistochemistry. A final diagnosis of iMCD was confirmed by the absence of the latter. The pathophysiology of CD is not fully understood. An increase in vascular endothelial growth factor and IL-6, although non-diagnostic, has been demonstrated and seems to be an important component of the cytokine storm seen in the disease.

The prognosis of individuals with iMCD is poor. The five-year and ten-year mortality rate is approximately 23-45% and 60%, respectively [18,19]. First-line treatment for iMCD is siltuximab, an anti-IL-6 monoclonal antibody that binds to and neutralizes IL-6, preventing it from binding to its receptors, thereby inhibiting IL-6-mediated signaling. This reduces inflammation and inhibits the formation of plasma cells and B lymphocytes, two important elements in the pathophysiology of CD [11,20]. Tocilizumab remains an alternative therapy and unlike siltuximab, which binds directly to IL-6, tocilizumab binds to the IL-6 receptor [21]. Chemotherapy may be used if the response is insufficient [10]. Close clinical follow-up is imperative to determine treatment response, disease progression, and complication monitoring [7].

## Conclusions

iMCD presents diagnostic and therapeutic challenges due to its rarity and diverse clinical manifestations. A comprehensive approach integrating clinical, radiological, and histopathological assessments is needed to reach an accurate diagnosis. The CDCN criteria aid in the diagnosis, emphasizing major and minor criteria alongside exclusions. Elevated IL-6 levels and characteristic histopathological features facilitate the diagnosis, although definitive confirmation often requires the exclusion of other diseases. Management focuses on controlling symptoms, reducing systemic inflammation, and halting disease progression, primarily through IL-6 blockade. The prognosis remains poor, necessitating close monitoring and prompt intervention. Further research is warranted to elucidate the pathophysiology of iMCD and refine treatment strategies to improve patient outcomes.
